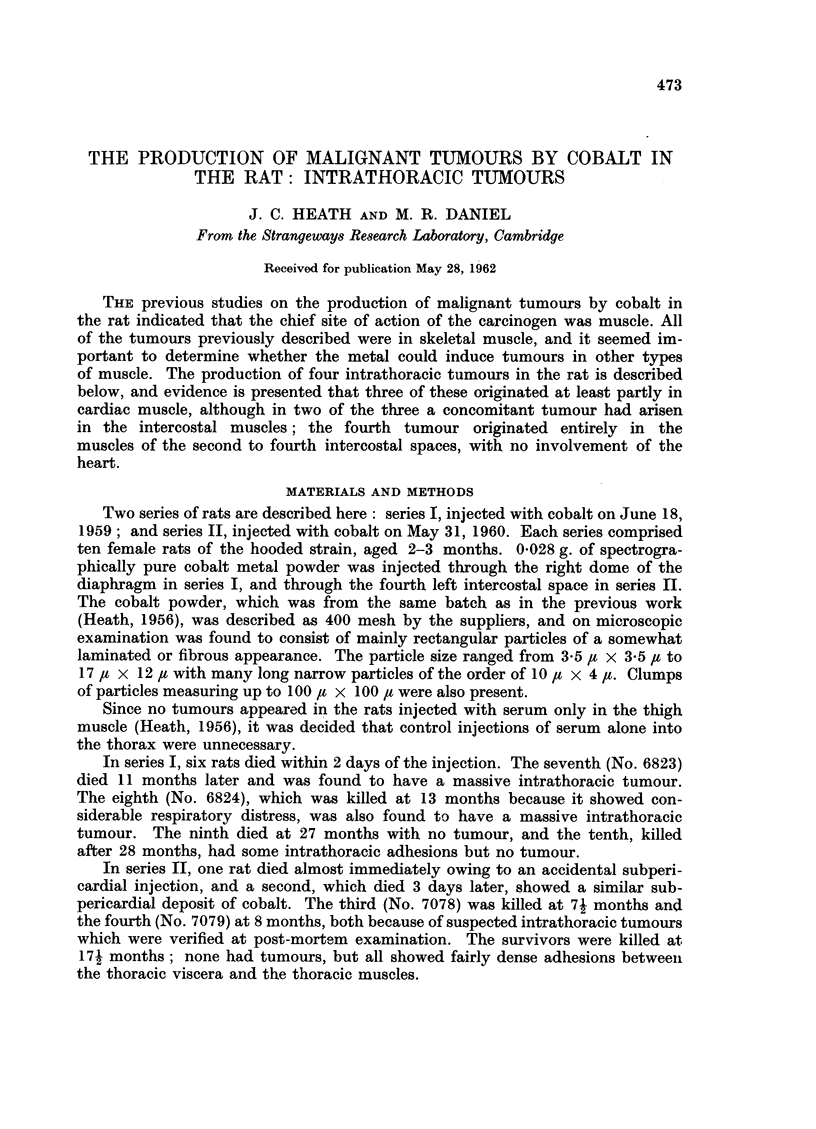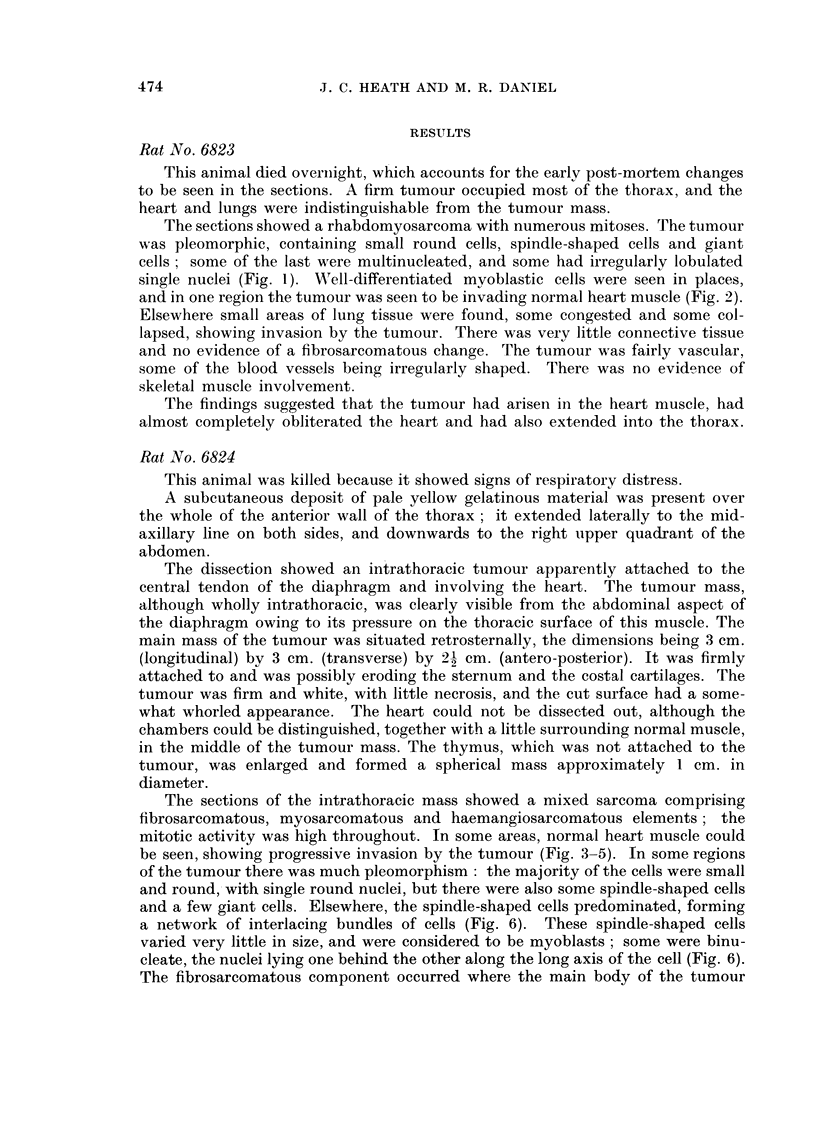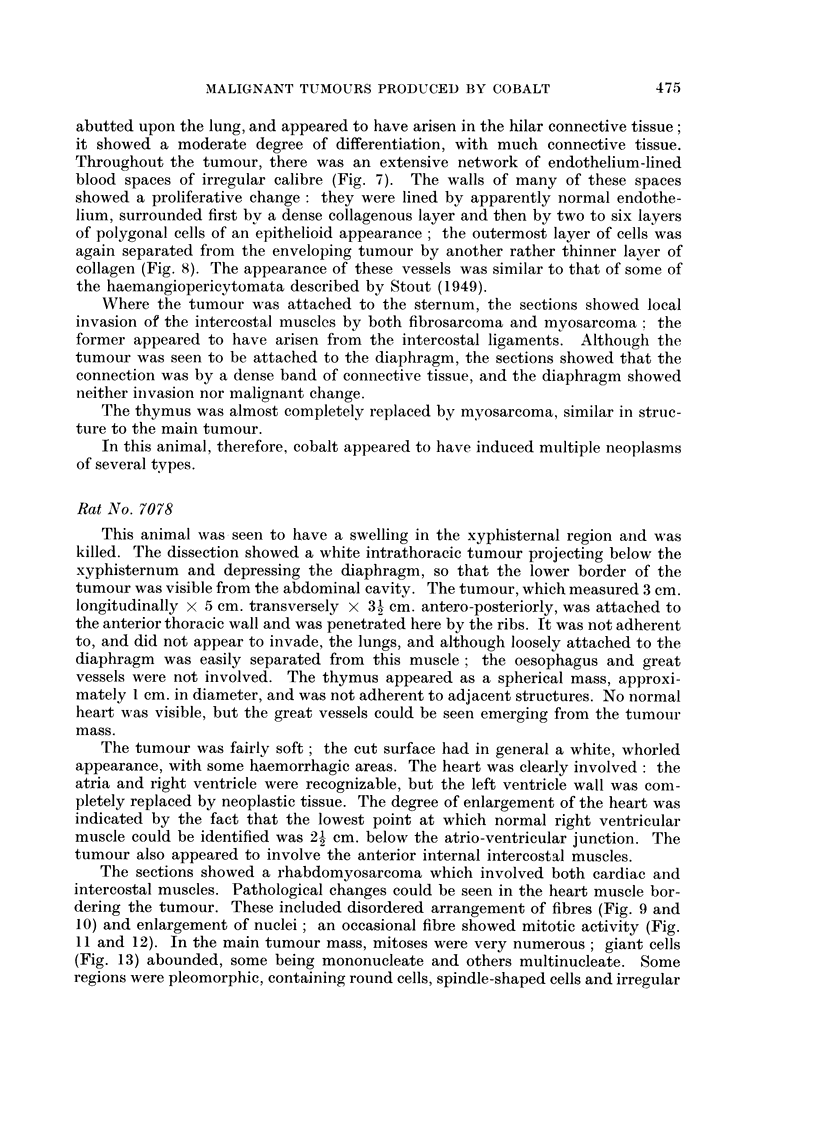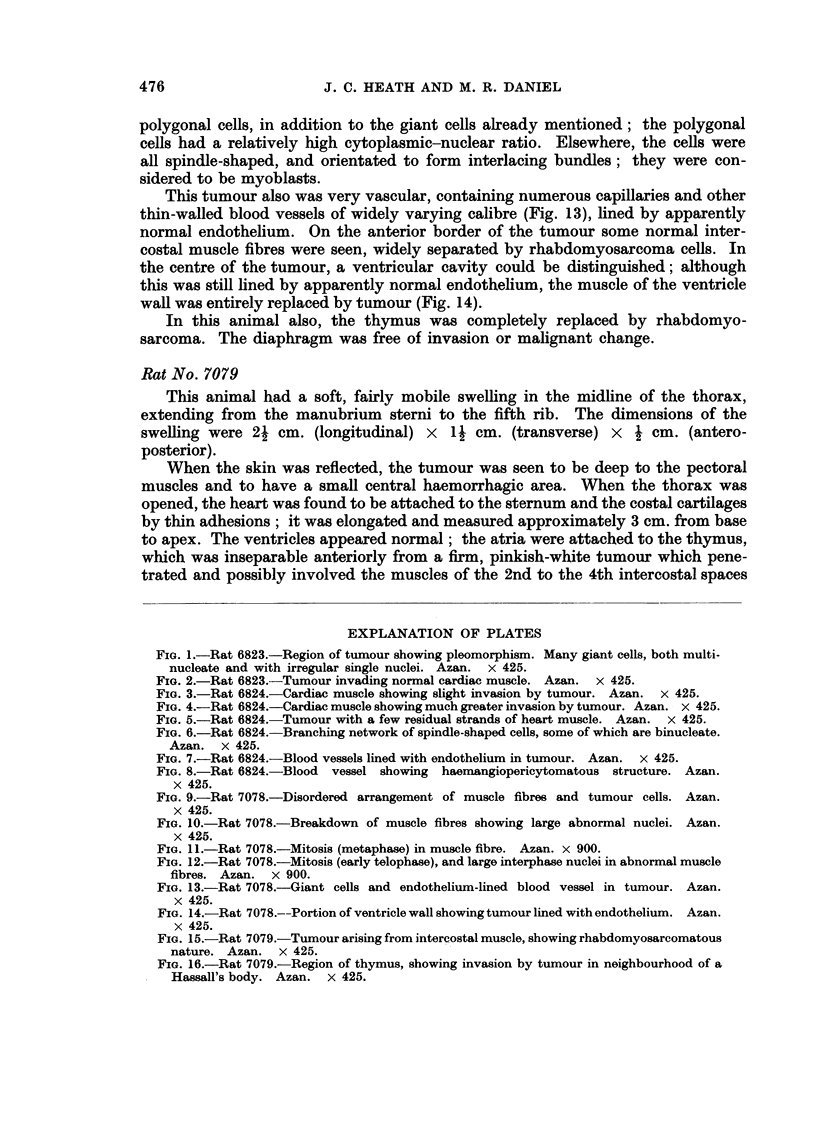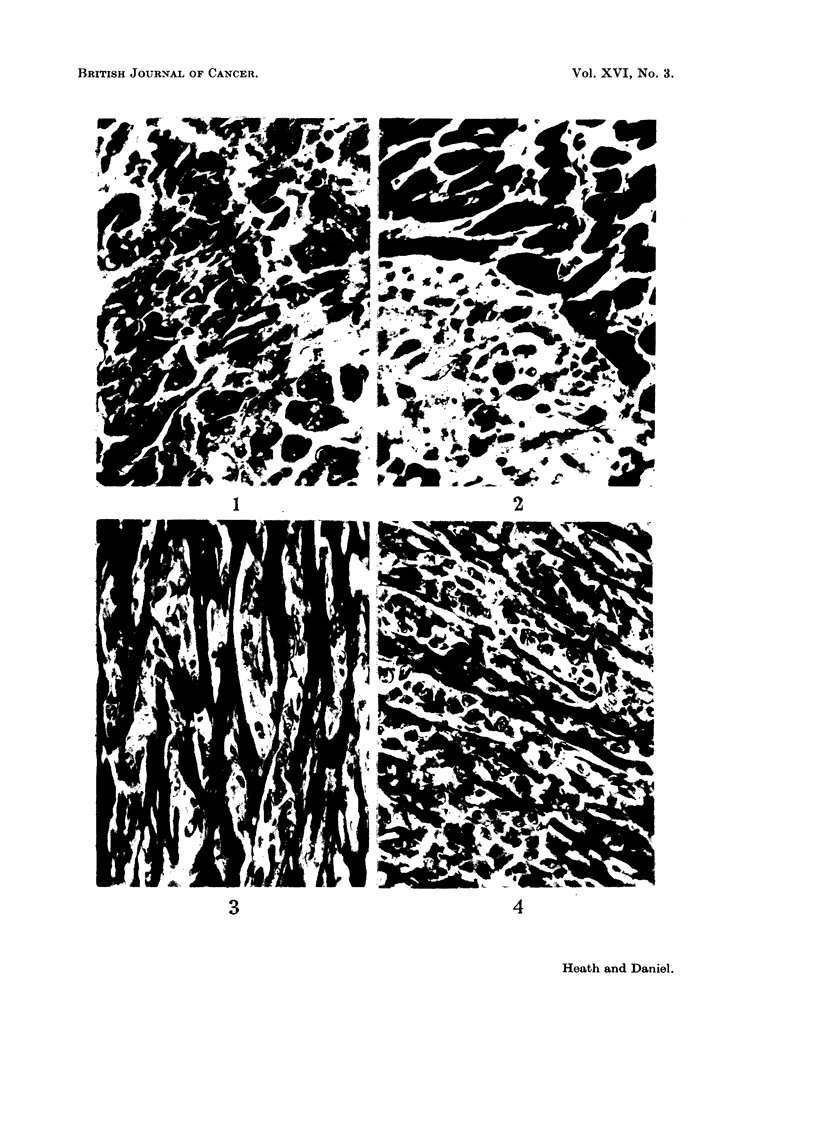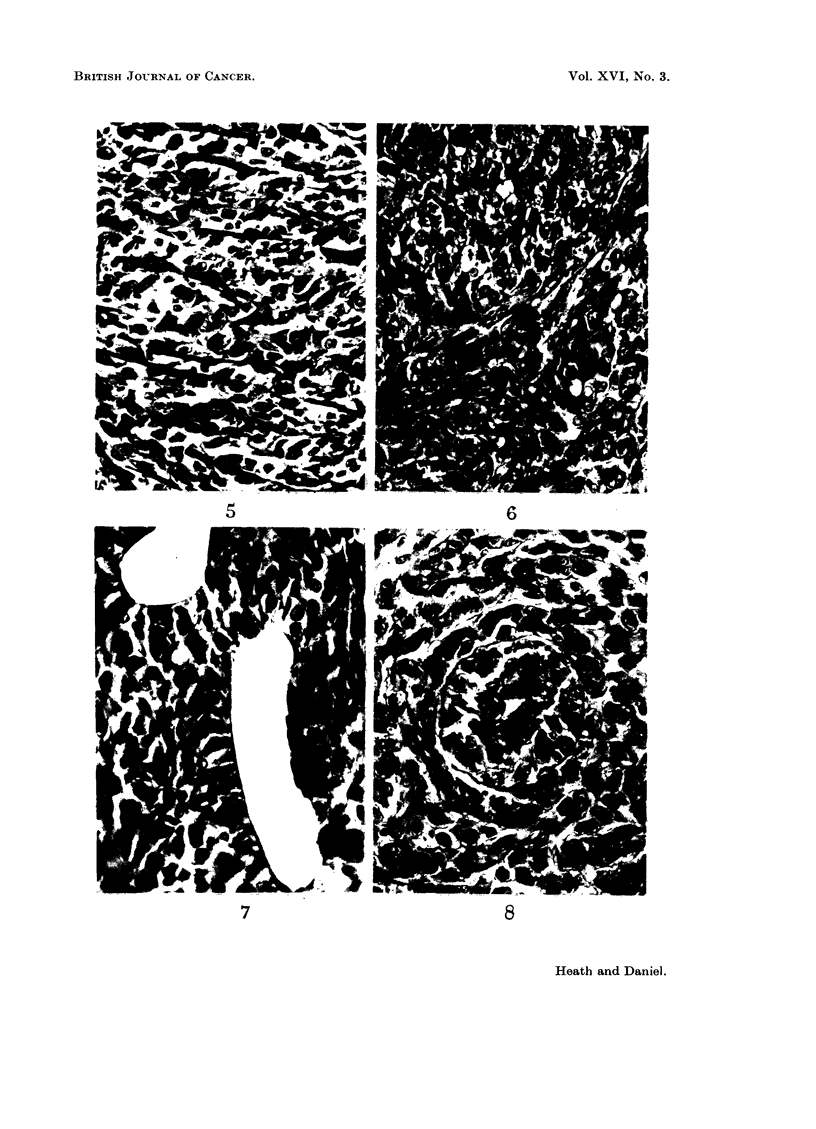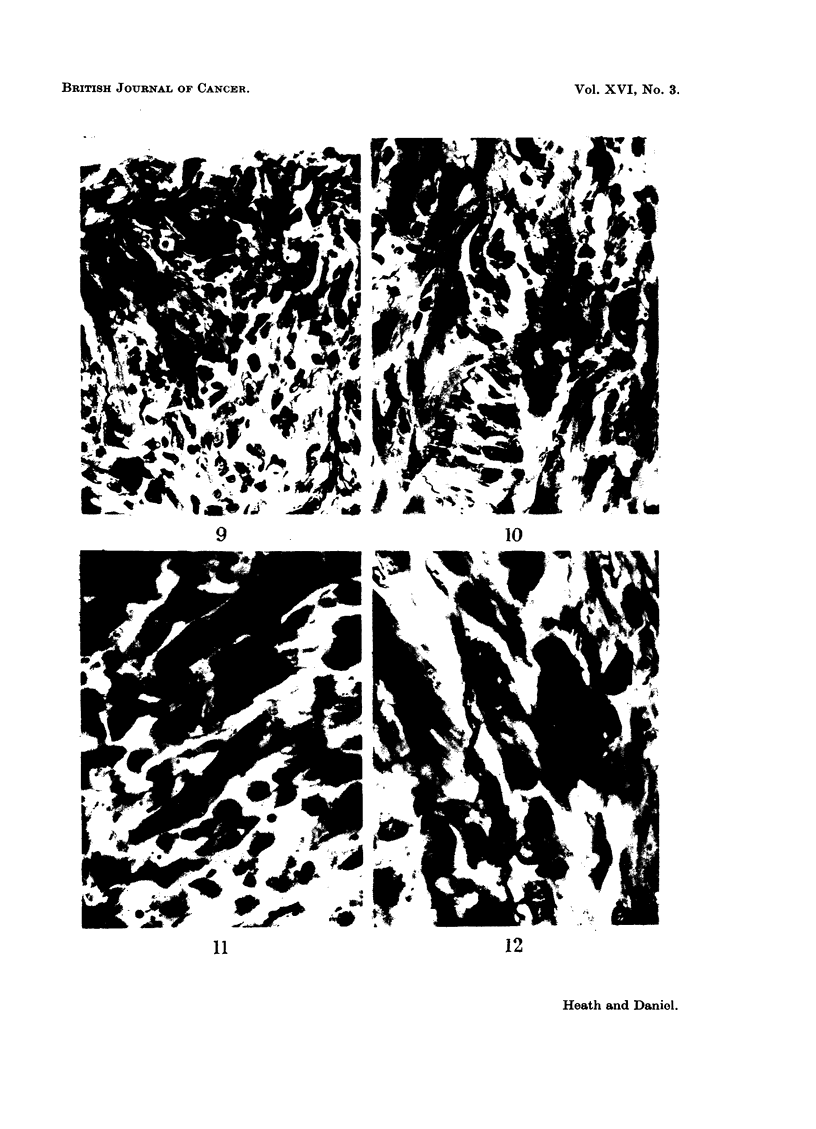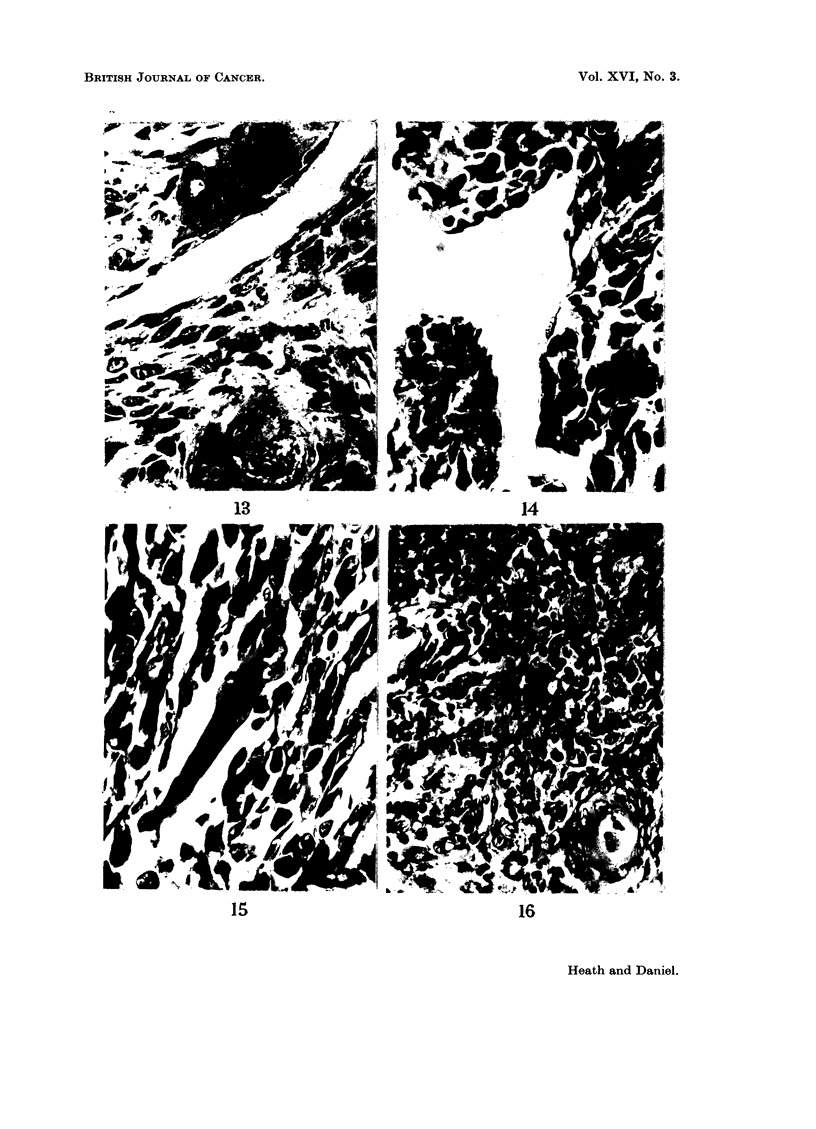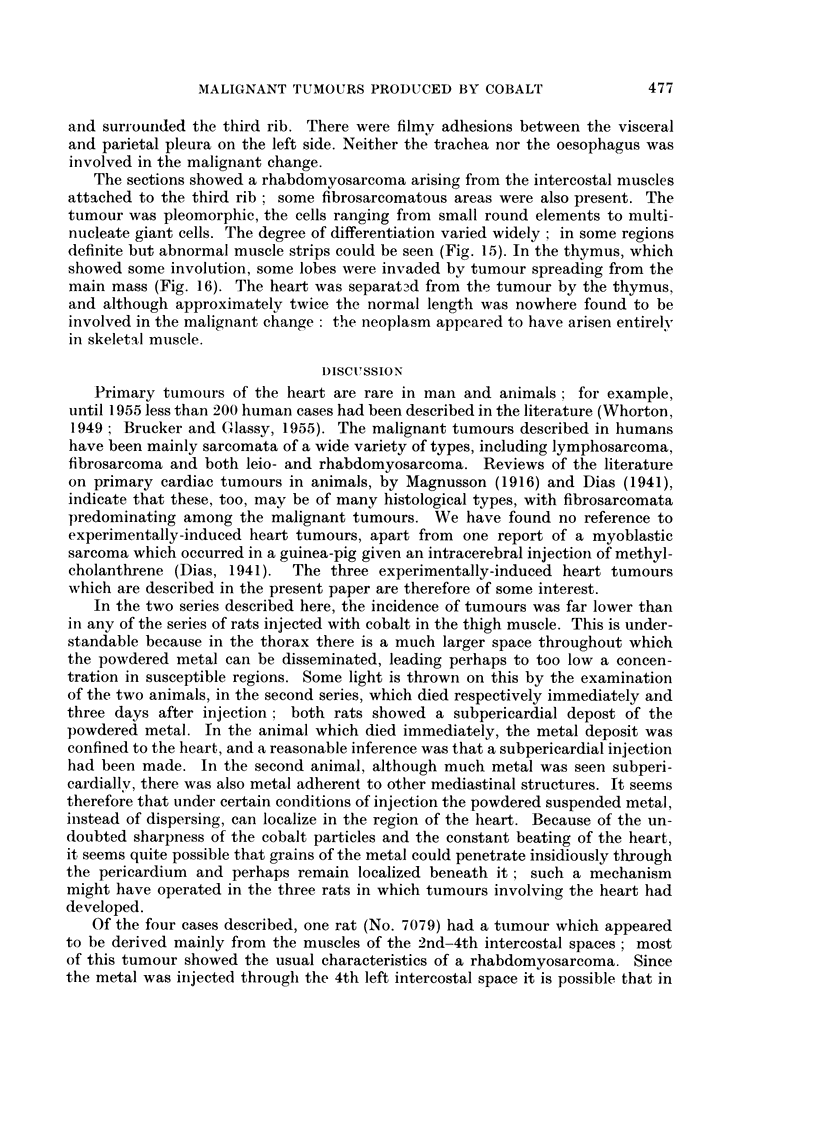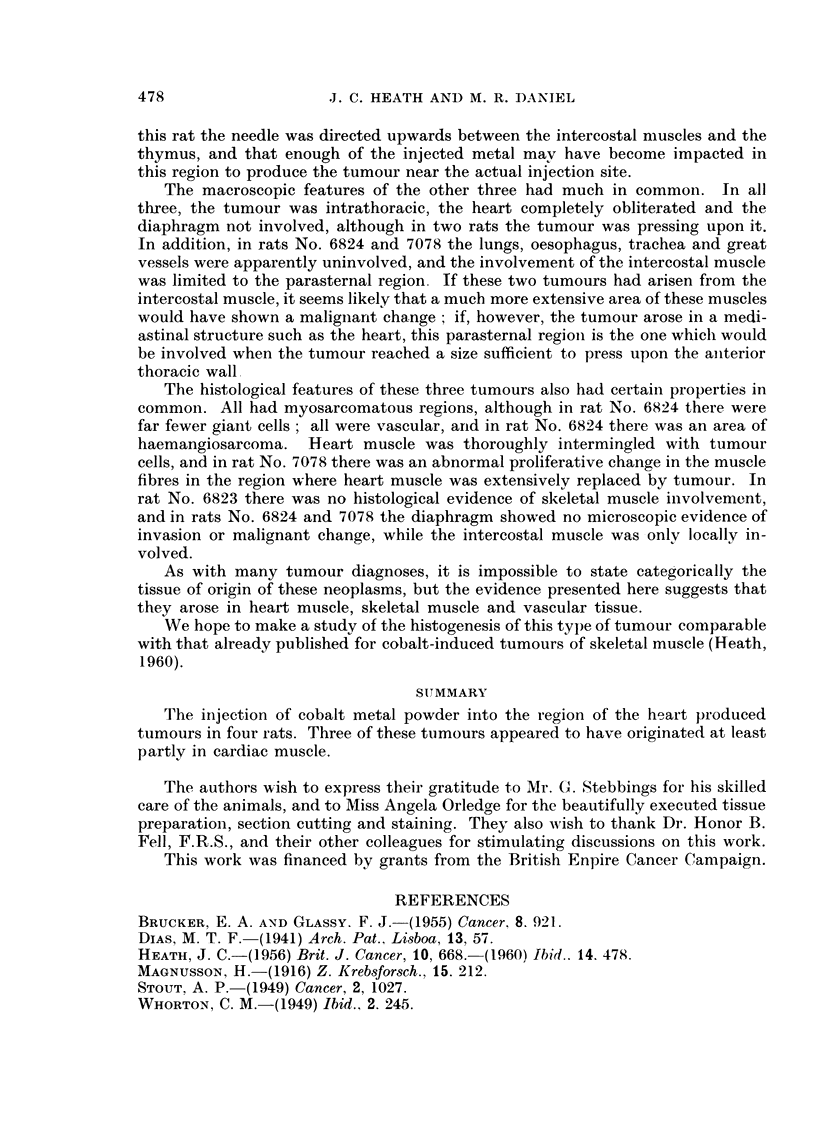# The Production of Malignant Tumours by Cobalt in the Rat: Intrathoracic Tumours

**DOI:** 10.1038/bjc.1962.53

**Published:** 1962-09

**Authors:** J. C. Heath, M. R. Daniel

## Abstract

**Images:**


### THE PRODUCTION OF MALIGNANT TUMOURS BY COBALT IN THE RAT: INTRATHORACIC TUMOURS

J. C. HEATH AND M. R. DANIEL

*From the Strangeways Research Laboratory, Cambridge*

Received for publication May 28, 1962

THE previous studies on the production of malignant tumours by cobalt in the rat indicated that the chief site of action of the carcinogen was muscle. All of the tumours previously described were in skeletal muscle, and it seemed important to determine whether the metal could induce tumours in other types of muscle. The production of four intrathoracic tumours in the rat is described below, and evidence is presented that three of these originated at least partly in cardiac muscle, although in two of the three a concomitant tumour had arisen in the intercostal muscles; the fourth tumour originated entirely in the muscles of the second to fourth intercostal spaces, with no involvement of the heart.

### MATERIALS AND METHODS

Two series of rats are described here: series I, injected with cobalt on June 18, 1959; and series II, injected with cobalt on May 31, 1960. Each series comprised ten female rats of the hooded strain, aged 2–3 months. 0·028 g. of spectrographically pure cobalt metal powder was injected through the right dome of the diaphragm in series I, and through the fourth left intercostal space in series II. The cobalt powder, which was from the same batch as in the previous work (Heath, 1956), was described as 400 mesh by the suppliers, and on microscopic examination was found to consist of mainly rectangular particles of a somewhat laminated or fibrous appearance. The particle size ranged from $3{\cdot}5\ \mu \times 3{\cdot}5\ \mu$ to $17\ \mu \times 12\ \mu$ with many long narrow particles of the order of $10\ \mu \times 4\ \mu$. Clumps of particles measuring up to $100\ \mu \times 100\ \mu$ were also present.

Since no tumours appeared in the rats injected with serum only in the thigh muscle (Heath, 1956), it was decided that control injections of serum alone into the thorax were unnecessary.

In series I, six rats died within 2 days of the injection. The seventh (No. 6823) died 11 months later and was found to have a massive intrathoracic tumour. The eighth (No. 6824), which was killed at 13 months because it showed considerable respiratory distress, was also found to have a massive intrathoracic tumour. The ninth died at 27 months with no tumour, and the tenth, killed after 28 months, had some intrathoracic adhesions but no tumour.

In series II, one rat died almost immediately owing to an accidental subpericardial injection, and a second, which died 3 days later, showed a similar subpericardial deposit of cobalt. The third (No. 7078) was killed at 7½ months and the fourth (No. 7079) at 8 months, both because of suspected intrathoracic tumours which were verified at post-mortem examination. The survivors were killed at 17½ months; none had tumours, but all showed fairly dense adhesions between the thoracic viscera and the thoracic muscles.

### RESULTS

*Rat No. 6823*

This animal died overnight, which accounts for the early post-mortem changes to be seen in the sections. A firm tumour occupied most of the thorax, and the heart and lungs were indistinguishable from the tumour mass.

The sections showed a rhabdomyosarcoma with numerous mitoses. The tumour was pleomorphic, containing small round cells, spindle-shaped cells and giant cells ; some of the last were multinucleated, and some had irregularly lobulated single nuclei (Fig. 1). Well-differentiated myoblastic cells were seen in places, and in one region the tumour was seen to be invading normal heart muscle (Fig. 2). Elsewhere small areas of lung tissue were found, some congested and some collapsed, showing invasion by the tumour. There was very little connective tissue and no evidence of a fibrosarcomatous change. The tumour was fairly vascular, some of the blood vessels being irregularly shaped. There was no evidence of skeletal muscle involvement.

The findings suggested that the tumour had arisen in the heart muscle, had almost completely obliterated the heart and had also extended into the thorax.

*Rat No. 6824*

This animal was killed because it showed signs of respiratory distress.

A subcutaneous deposit of pale yellow gelatinous material was present over the whole of the anterior wall of the thorax ; it extended laterally to the mid-axillary line on both sides, and downwards to the right upper quadrant of the abdomen.

The dissection showed an intrathoracic tumour apparently attached to the central tendon of the diaphragm and involving the heart. The tumour mass, although wholly intrathoracic, was clearly visible from the abdominal aspect of the diaphragm owing to its pressure on the thoracic surface of this muscle. The main mass of the tumour was situated retrosternally, the dimensions being 3 cm. (longitudinal) by 3 cm. (transverse) by $2\frac{1}{2}$ cm. (antero-posterior). It was firmly attached to and was possibly eroding the sternum and the costal cartilages. The tumour was firm and white, with little necrosis, and the cut surface had a somewhat whorled appearance. The heart could not be dissected out, although the chambers could be distinguished, together with a little surrounding normal muscle, in the middle of the tumour mass. The thymus, which was not attached to the tumour, was enlarged and formed a spherical mass approximately 1 cm. in diameter.

The sections of the intrathoracic mass showed a mixed sarcoma comprising fibrosarcomatous, myosarcomatous and haemangiosarcomatous elements ; the mitotic activity was high throughout. In some areas, normal heart muscle could be seen, showing progressive invasion by the tumour (Fig. 3–5). In some regions of the tumour there was much pleomorphism : the majority of the cells were small and round, with single round nuclei, but there were also some spindle-shaped cells and a few giant cells. Elsewhere, the spindle-shaped cells predominated, forming a network of interlacing bundles of cells (Fig. 6). These spindle-shaped cells varied very little in size, and were considered to be myoblasts ; some were binucleate, the nuclei lying one behind the other along the long axis of the cell (Fig. 6). The fibrosarcomatous component occurred where the main body of the tumour abutted upon the lung, and appeared to have arisen in the hilar connective tissue; it showed a moderate degree of differentiation, with much connective tissue. Throughout the tumour, there was an extensive network of endothelium-lined blood spaces of irregular calibre (Fig. 7). The walls of many of these spaces showed a proliferative change : they were lined by apparently normal endothelium, surrounded first by a dense collagenous layer and then by two to six layers of polygonal cells of an epithelioid appearance ; the outermost layer of cells was again separated from the enveloping tumour by another rather thinner layer of collagen (Fig. 8). The appearance of these vessels was similar to that of some of the haemangiopericytomata described by Stout (1949).

Where the tumour was attached to the sternum, the sections showed local invasion of the intercostal muscles by both fibrosarcoma and myosarcoma ; the former appeared to have arisen from the intercostal ligaments. Although the tumour was seen to be attached to the diaphragm, the sections showed that the connection was by a dense band of connective tissue, and the diaphragm showed neither invasion nor malignant change.

The thymus was almost completely replaced by myosarcoma, similar in structure to the main tumour.

In this animal, therefore, cobalt appeared to have induced multiple neoplasms of several types.

*Rat No. 7078*

This animal was seen to have a swelling in the xyphisternal region and was killed. The dissection showed a white intrathoracic tumour projecting below the xyphisternum and depressing the diaphragm, so that the lower border of the tumour was visible from the abdominal cavity. The tumour, which measured 3 cm. longitudinally $\times$ 5 cm. transversely $\times$ $3\frac{1}{2}$ cm. antero-posteriorly, was attached to the anterior thoracic wall and was penetrated here by the ribs. It was not adherent to, and did not appear to invade, the lungs, and although loosely attached to the diaphragm was easily separated from this muscle ; the oesophagus and great vessels were not involved. The thymus appeared as a spherical mass, approximately 1 cm. in diameter, and was not adherent to adjacent structures. No normal heart was visible, but the great vessels could be seen emerging from the tumour mass.

The tumour was fairly soft ; the cut surface had in general a white, whorled appearance, with some haemorrhagic areas. The heart was clearly involved : the atria and right ventricle were recognizable, but the left ventricle wall was completely replaced by neoplastic tissue. The degree of enlargement of the heart was indicated by the fact that the lowest point at which normal right ventricular muscle could be identified was $2\frac{1}{2}$ cm. below the atrio-ventricular junction. The tumour also appeared to involve the anterior internal intercostal muscles.

The sections showed a rhabdomyosarcoma which involved both cardiac and intercostal muscles. Pathological changes could be seen in the heart muscle bordering the tumour. These included disordered arrangement of fibres (Fig. 9 and 10) and enlargement of nuclei ; an occasional fibre showed mitotic activity (Fig. 11 and 12). In the main tumour mass, mitoses were very numerous ; giant cells (Fig. 13) abounded, some being mononucleate and others multinucleate. Some regions were pleomorphic, containing round cells, spindle-shaped cells and irregular polygonal cells, in addition to the giant cells already mentioned ; the polygonal cells had a relatively high cytoplasmic–nuclear ratio. Elsewhere, the cells were all spindle-shaped, and orientated to form interlacing bundles ; they were considered to be myoblasts.

This tumour also was very vascular, containing numerous capillaries and other thin-walled blood vessels of widely varying calibre (Fig. 13), lined by apparently normal endothelium. On the anterior border of the tumour some normal intercostal muscle fibres were seen, widely separated by rhabdomyosarcoma cells. In the centre of the tumour, a ventricular cavity could be distinguished; although this was still lined by apparently normal endothelium, the muscle of the ventricle wall was entirely replaced by tumour (Fig. 14).

In this animal also, the thymus was completely replaced by rhabdomyosarcoma. The diaphragm was free of invasion or malignant change.

*Rat No. 7079*

This animal had a soft, fairly mobile swelling in the midline of the thorax, extending from the manubrium sterni to the fifth rib. The dimensions of the swelling were $2\frac{1}{2}$ cm. (longitudinal) $\times$ $1\frac{1}{2}$ cm. (transverse) $\times$ $\frac{1}{2}$ cm. (antero-posterior).

When the skin was reflected, the tumour was seen to be deep to the pectoral muscles and to have a small central haemorrhagic area. When the thorax was opened, the heart was found to be attached to the sternum and the costal cartilages by thin adhesions ; it was elongated and measured approximately 3 cm. from base to apex. The ventricles appeared normal ; the atria were attached to the thymus, which was inseparable anteriorly from a firm, pinkish-white tumour which penetrated and possibly involved the muscles of the 2nd to the 4th intercostal spaces

EXPLANATION OF PLATES

FIG. 1.—Rat 6823.—Region of tumour showing pleomorphism. Many giant cells, both multinucleate and with irregular single nuclei. Azan. × 425.
FIG. 2.—Rat 6823.—Tumour invading normal cardiac muscle. Azan. × 425.
FIG. 3.—Rat 6824.—Cardiac muscle showing slight invasion by tumour. Azan. × 425.
FIG. 4.—Rat 6824.—Cardiac muscle showing much greater invasion by tumour. Azan. × 425.
FIG. 5.—Rat 6824.—Tumour with a few residual strands of heart muscle. Azan. × 425.
FIG. 6.—Rat 6824.—Branching network of spindle-shaped cells, some of which are binucleate. Azan. × 425.
FIG. 7.—Rat 6824.—Blood vessels lined with endothelium in tumour. Azan. × 425.
FIG. 8.—Rat 6824.—Blood vessel showing haemangiopericytomatous structure. Azan. × 425.
FIG. 9.—Rat 7078.—Disordered arrangement of muscle fibres and tumour cells. Azan. × 425.
FIG. 10.—Rat 7078.—Breakdown of muscle fibres showing large abnormal nuclei. Azan. × 425.
FIG. 11.—Rat 7078.—Mitosis (metaphase) in muscle fibre. Azan. × 900.
FIG. 12.—Rat 7078.—Mitosis (early telophase), and large interphase nuclei in abnormal muscle fibres. Azan. × 900.
FIG. 13.—Rat 7078.—Giant cells and endothelium-lined blood vessel in tumour. Azan. × 425.
FIG. 14.—Rat 7078.—Portion of ventricle wall showing tumour lined with endothelium. Azan. × 425.
FIG. 15.—Rat 7079.—Tumour arising from intercostal muscle, showing rhabdomyosarcomatous nature. Azan. × 425.
FIG. 16.—Rat 7079.—Region of thymus, showing invasion by tumour in neighbourhood of a Hassall's body. Azan. × 425.

1 2 3 4

Heath and Daniel.

5

6

7

8

Heath and Daniel.

9

10

11

12

Heath and Daniel.

13

14

15

16

Heath and Daniel.

and surrounded the third rib. There were filmy adhesions between the visceral and parietal pleura on the left side. Neither the trachea nor the oesophagus was involved in the malignant change.

The sections showed a rhabdomyosarcoma arising from the intercostal muscles attached to the third rib ; some fibrosarcomatous areas were also present. The tumour was pleomorphic, the cells ranging from small round elements to multinucleate giant cells. The degree of differentiation varied widely ; in some regions definite but abnormal muscle strips could be seen (Fig. 15). In the thymus, which showed some involution, some lobes were invaded by tumour spreading from the main mass (Fig. 16). The heart was separated from the tumour by the thymus, and although approximately twice the normal length was nowhere found to be involved in the malignant change : the neoplasm appeared to have arisen entirely in skeletal muscle.

### DISCUSSION

Primary tumours of the heart are rare in man and animals ; for example, until 1955 less than 200 human cases had been described in the literature (Whorton, 1949 ; Brucker and Glassy, 1955). The malignant tumours described in humans have been mainly sarcomata of a wide variety of types, including lymphosarcoma, fibrosarcoma and both leio- and rhabdomyosarcoma. Reviews of the literature on primary cardiac tumours in animals, by Magnusson (1916) and Dias (1941), indicate that these, too, may be of many histological types, with fibrosarcomata predominating among the malignant tumours. We have found no reference to experimentally-induced heart tumours, apart from one report of a myoblastic sarcoma which occurred in a guinea-pig given an intracerebral injection of methylcholanthrene (Dias, 1941). The three experimentally-induced heart tumours which are described in the present paper are therefore of some interest.

In the two series described here, the incidence of tumours was far lower than in any of the series of rats injected with cobalt in the thigh muscle. This is understandable because in the thorax there is a much larger space throughout which the powdered metal can be disseminated, leading perhaps to too low a concentration in susceptible regions. Some light is thrown on this by the examination of the two animals, in the second series, which died respectively immediately and three days after injection ; both rats showed a subpericardial depost of the powdered metal. In the animal which died immediately, the metal deposit was confined to the heart, and a reasonable inference was that a subpericardial injection had been made. In the second animal, although much metal was seen subpericardially, there was also metal adherent to other mediastinal structures. It seems therefore that under certain conditions of injection the powdered suspended metal, instead of dispersing, can localize in the region of the heart. Because of the undoubted sharpness of the cobalt particles and the constant beating of the heart, it seems quite possible that grains of the metal could penetrate insidiously through the pericardium and perhaps remain localized beneath it ; such a mechanism might have operated in the three rats in which tumours involving the heart had developed.

Of the four cases described, one rat (No. 7079) had a tumour which appeared to be derived mainly from the muscles of the 2nd–4th intercostal spaces ; most of this tumour showed the usual characteristics of a rhabdomyosarcoma. Since the metal was injected through the 4th left intercostal space it is possible that in this rat the needle was directed upwards between the intercostal muscles and the thymus, and that enough of the injected metal may have become impacted in this region to produce the tumour near the actual injection site.

The macroscopic features of the other three had much in common. In all three, the tumour was intrathoracic, the heart completely obliterated and the diaphragm not involved, although in two rats the tumour was pressing upon it. In addition, in rats No. 6824 and 7078 the lungs, oesophagus, trachea and great vessels were apparently uninvolved, and the involvement of the intercostal muscle was limited to the parasternal region. If these two tumours had arisen from the intercostal muscle, it seems likely that a much more extensive area of these muscles would have shown a malignant change ; if, however, the tumour arose in a mediastinal structure such as the heart, this parasternal region is the one which would be involved when the tumour reached a size sufficient to press upon the anterior thoracic wall.

The histological features of these three tumours also had certain properties in common. All had myosarcomatous regions, although in rat No. 6824 there were far fewer giant cells ; all were vascular, and in rat No. 6824 there was an area of haemangiosarcoma. Heart muscle was thoroughly intermingled with tumour cells, and in rat No. 7078 there was an abnormal proliferative change in the muscle fibres in the region where heart muscle was extensively replaced by tumour. In rat No. 6823 there was no histological evidence of skeletal muscle involvement, and in rats No. 6824 and 7078 the diaphragm showed no microscopic evidence of invasion or malignant change, while the intercostal muscle was only locally involved.

As with many tumour diagnoses, it is impossible to state categorically the tissue of origin of these neoplasms, but the evidence presented here suggests that they arose in heart muscle, skeletal muscle and vascular tissue.

We hope to make a study of the histogenesis of this type of tumour comparable with that already published for cobalt-induced tumours of skeletal muscle (Heath, 1960).

### SUMMARY

The injection of cobalt metal powder into the region of the heart produced tumours in four rats. Three of these tumours appeared to have originated at least partly in cardiac muscle.

The authors wish to express their gratitude to Mr. G. Stebbings for his skilled care of the animals, and to Miss Angela Orledge for the beautifully executed tissue preparation, section cutting and staining. They also wish to thank Dr. Honor B. Fell, F.R.S., and their other colleagues for stimulating discussions on this work.

This work was financed by grants from the British Enpire Cancer Campaign.

### REFERENCES

BRUCKER, E. A. AND GLASSY, F. J.—(1955) *Cancer*, **8**, 921.
DIAS, M. T. F.—(1941) *Arch. Pat., Lisboa*, **13**, 57.
HEATH, J. C.—(1956) *Brit. J. Cancer*, **10**, 668.—(1960) *Ibid.*, **14**, 478.
MAGNUSSON, H.—(1916) *Z. Krebsforsch.*, **15**, 212.
STOUT, A. P.—(1949) *Cancer*, **2**, 1027.
WHORTON, C. M.—(1949) *Ibid.*, **2**, 245.